# Vitamin D attenuates myofibroblast differentiation and extracellular matrix accumulation in nasal polyp-derived fibroblasts through smad2/3 signaling pathway

**DOI:** 10.1038/s41598-017-07561-6

**Published:** 2017-08-04

**Authors:** Seoung-Ae Lee, Hyun-Woo Yang, Ji-Young Um, Jae-Min Shin, Il-Ho Park, Heung-Man Lee

**Affiliations:** 1 Institute for Medical Devices Clinical Trial Center, Korea University Guro Hospital, Korea University College of Medicine, Seoul, South Korea; 2 Department of Biomedical Science, Korea University College of Medicine, Seoul, South Korea; 3 Department of Otorhinolaryngology-Head and Neck Surgery, Korea University College of Medicine, Seoul, South Korea; 4 Research-Driven Hospital, Korea University Guro Hospital, Korea University College of Medicine, Seoul, South Korea

## Abstract

To investigate the potential role of vitamin D (1,25(OH)_2_D_3_) in preventing the development of nasal polyps, we examined the effect of vitamin D on myofibroblast differentiation and extracellular matrix (ECM) production in TGF-β1-induced nasal polyp-derived fibroblasts (NPDFs) and elucidated the mechanisms underlying its inhibitory effect. 1,25(OH)_2_D_3_ significantly reduced expression levels of α-SMA, a myofibroblast marker, and fibronectin, a representative ECM component, in a dose-dependent manner in TGF-β1-induced NPDFs. 1,25(OH)_2_D_3_ suppressed activated Smad2/3 in time-course. Up-regulation of α-SMA, fibronectin and phosphorylation of Smad2/3 by TGF-β1 was unaffected by 1,25(OH)_2_D_3_ in NPDFs after vitamin D receptor-specific siRNA transfection. We confirmed that the Smad2/3-specific inhibitor SIS3 inactivated Smad2/3 and reduced α-SMA and fibronectin expression. Furthermore, acetylation of histone H3 was compromised by 1,25(OH)_2_D_3,_ leading to inhibition of *collagen 1A1*, *collagen 1A2* and *α-SMA* gene expression. Treatment with 1,25(OH)_2_D_3_ also significantly suppressed TGF-β1-enhanced contractility and motility in a contraction assay and Transwell migration assay. Finally, 1,25(OH)_2_D_3_ had a similar effect in *ex vivo* organ cultures of nasal polyps. Taken together, our results suggest that 1,25(OH)_2_D_3_ might be an effective therapy for nasal polyps by reducing myofibroblast differentiation and ECM production mediated by Smad2/3-dependent TGF-β1 signaling pathways in NPDFs.

## Introduction

Chronic rhinosinusitis with nasal polyps (CRSwNP) is a growing public health problem affecting between 1% and 4% of the world population, and new pharmacological agents are needed to combat this disease^1, 2^. Nasal polyp, a common inflammatory disease of the nasal and paranasal mucosa, is characterized by inflammatory cell accumulation, basement membrane thickening, abnormal proliferation of fibroblasts, and exaggerated deposition of extracellular matrix (ECM)^3, 4^.

When nasal polyps develop, fibroblasts transform into myofibroblasts that express α- smooth muscle actin (α*-*SMA) and subsequently overproduce ECM components such as glycosaminoglycans, fibronectins, and collagen types I, IV, VI, and VII. Therefore, fibroblasts are target cells for the treatment of nasal polyps and may serve an important function in the process of nasal polyp formation by blocking major factors of cell differentiation and ECM production^5^.

Vitamin D is synthesized in the skin or consumed via nutritional sources and modulates bone development and calcium homeostasis^6^. However, recent reports have shown that vitamin D also has a wide range of antifibrotic properties, including anti-inflammation, anti-proliferation, anti-apoptosis, and anti-epithelial-mesenchymal transition properties^7–10^. Several documents have shown that vitamin D deficiency is associated with the severity of asthma and the severity of bone erosion due to immune dysfunction in CRSwNP^11, 12^. Vitamin D taken during pregnancy may be adversely linked to increased risk of asthma and allergic rhinitis in childhood^13^. In addition, vitamin D derivatives were shown to inhibit matrix metalloproteinase (MMP)-2 and MMP-9 as well as eotaxin and regulated on activation, normal T cell expressed and secreted (RANTES) secretions in nasal polyp-derived fibroblasts (NPDFs) from Taiwanese patients with CRSwNP^14, 15^.

Little is known regarding the mechanisms involved in the anti-tissue remodeling effect of vitamin D in TGF-β1-induced NPDFs. TGF-β1-mediated activation of Smad signaling is responsible for tissue fibrosis and remodeling in several organs^16, 17^. In this study, we investigated whether vitamin D could prevent myofibroblast differentiation and extracellular matrix synthesis in NPDFs and in *ex vivo* organ culture of nasal polyps. Furthermore, we investigated the potential mechanisms involved in the effects of vitamin D for the treatment of nasal polyps.

## Results

### 1,25(OH)_2_D_3_ suppresses myofibroblast differentiation in nasal polyp-derived fibroblasts

To examine the effects of vitamin D on myofibroblast differentiation and ECM production in NPDFs, we established NPDFs from patients with nasal polyps. The purity of NPDFs was confirmed based on spindle-shaped cell morphology under microscopic observation and by staining for vimentin, Thy-1, and E-cadherin, which are used as fibroblast and epithelial markers (data not shown). The effect of 1,25(OH)_2_D_3_ on viability in NPDFs was analyzed using MTT assay after treatment for 72 hours; no significant toxicity was observed in NPDFs treated with 1,25(OH)_2_D_3_ in a dose-dependent manner up to 1,000 nM (Fig. S1). Since the maximal concentration of 1,25(OH)_2_D_3_ achievable in nasal tissue is unknown, 100 nM was chosen as the optimal dose based on an *in vitro* study of vitamin D in fibroblasts^18^.

**Figure 1:**
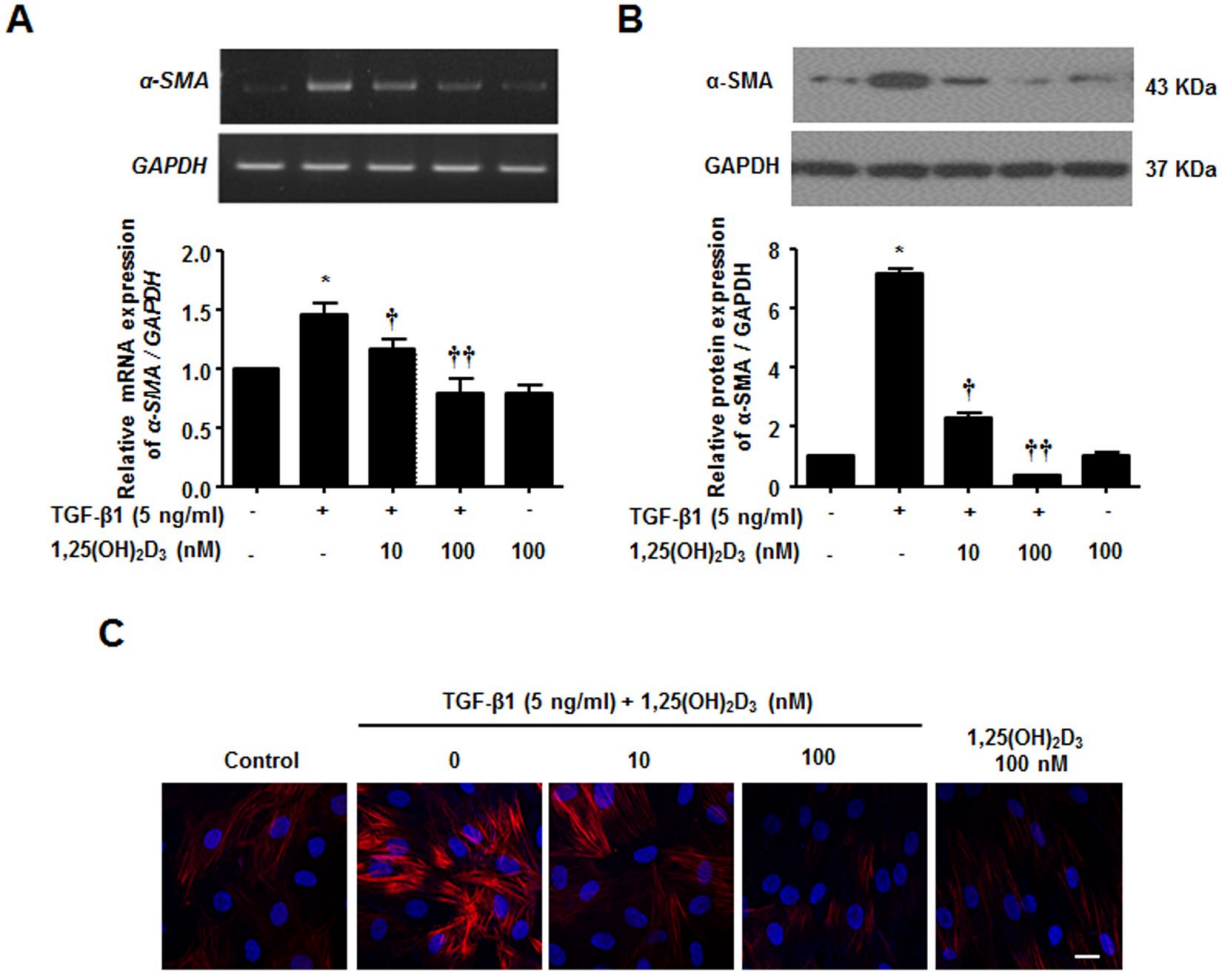
Inhibition of TGF-β1-induced α-SMA mRNA and protein expression by 1,25(OH)_2_D_3_ in nasal polyp-derived fibroblasts. Nasal polyp-derived fibroblasts were treated with TGF-β1 and/or 1,25(OH)_2_D_3_ or 1,25(OH)_2_D_3_ alone for 72 hours. (**A**,**B**) Expression levels of α-SMA mRNA and protein were determined by semi-quantitative RT-PCR analysis and Western blotting analysis. Expression of housekeeping GAPDH was used as an internal control. A representative experiment and quantitative determination of α-SMA mRNA and protein levels are shown. (**C**) Representative fluorescein immunocytochemistry for α-SMA (red) with nuclear DAPI (blue). Scale bar = 50 μm. All data are presented as mean ± SEM. Four primary cell lines from different donors were used. All experiments were performed in at least triplicate and repeated at least three times. *p < 0.05 vs. control, ^†^p < 0.05, ^††^p < 0.01 vs. TGF-β1.

α-SMA is a well-known marker of myofibroblast differentiation. α-SMA expression increased in TGF-β1-treated NPDFs compared to the control, in agreement with a previous report^19^. Given that 1,25(OH)_2_D_3_ was shown to suppress myofibroblast activation from interstitial fibroblasts^18^, we sought to confirm the inhibitory effect of 1,25(OH)_2_D_3_ in NPDFs. We found that α-SMA mRNA and protein expression levels were prevented by 1,25(OH)_2_D_3_ in a dose-dependent manner (Fig. 1A,B). Furthermore, immunofluorescence staining revealed a suppressive effect of 1,25(OH)_2_D_3_ on α-SMA expression; TGF-β1-stimulated NPDFs displayed abundant α-SMA expression in the cytoplasm (Fig. 1C). Taken together, these data demonstrate that 1,25(OH)_2_D_3_ reduced myofibroblast differentiation of TGF-β1-induced NPDFs, which was mediated by down-regulation of α-SMA.

### 1,25(OH)_2_D_3_ blocks TGF-β1-induced extracellular matrix production in nasal polyp-derived fibroblasts

**Figure 2:**
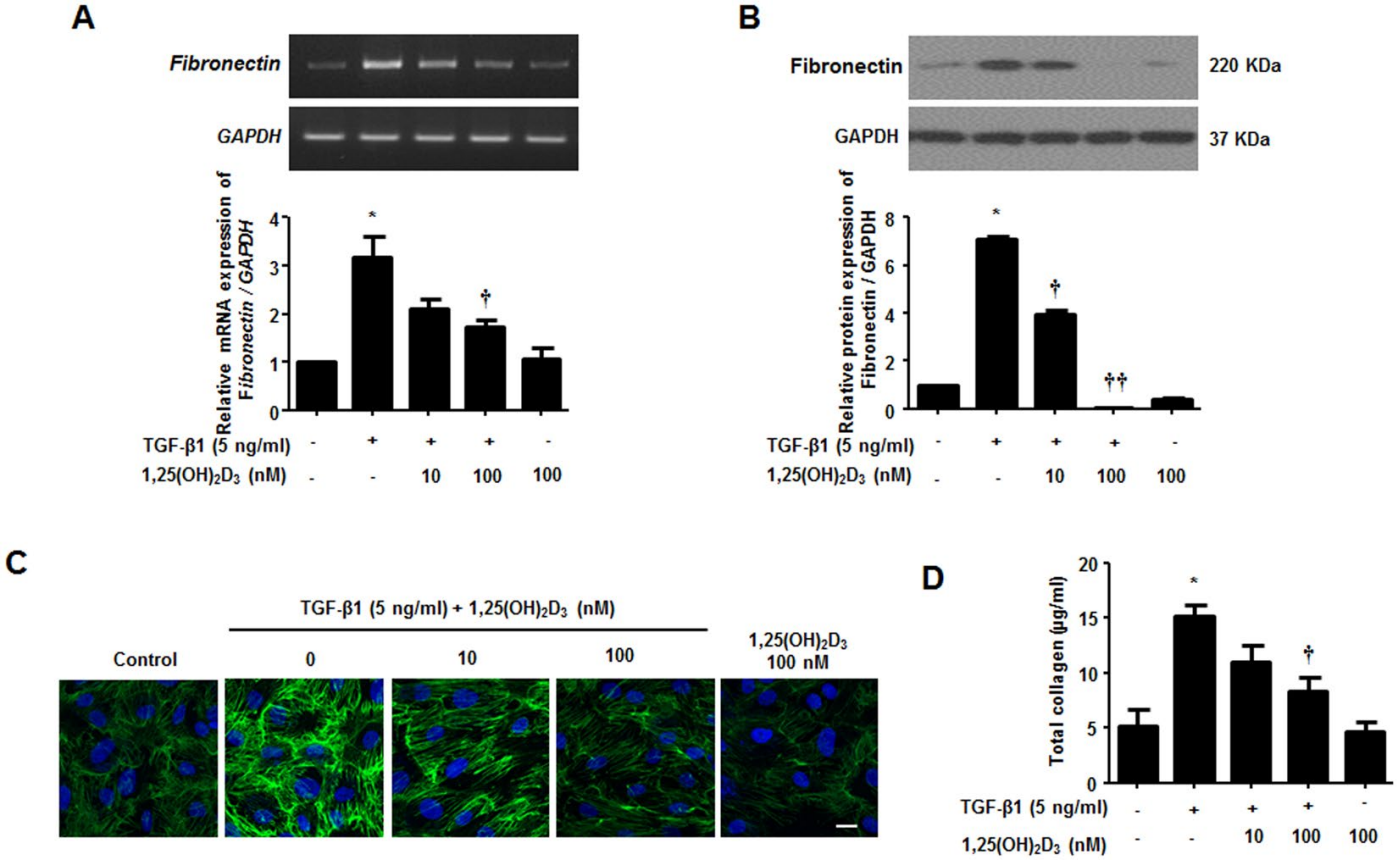
1,25(OH)_2_D_3_ decreases TGF-β1-induced collagen production in nasal polyp-derived fibroblasts. Nasal polyp-derived fibroblasts were treated with TGF-β1 and/or 1,25(OH)_2_D_3_ or 1,25(OH)_2_D_3_ alone for 72 hours. (**A**,**B**) Expression levels of fibronectin mRNA and protein were determined by semi-quantitative RT-PCR analysis and Western blot analysis. Expression levels of housekeeping GAPDH mRNA and protein were utilized as internal controls. A representative experiment and quantitative determination of fibronectin mRNA and protein levels are shown. (**C**) Representative fluorescein immunocytochemistry for fibronectin (green) with nuclear DAPI (blue). Scale bar = 50 μm. (**D**) The amount of total soluble collagen in culture media was quantified by the Sircol collagen assay. All data are presented as mean ± SEM. Four primary cell lines from different donors were used. All experiments were performed in at least triplicate and repeated at least three times. *p < 0.05 vs. control, ^†^p < 0.05, ^††^p < 0.01 vs. TGF-β1.

TGF-β1, a representative pro-fibrotic cytokine, induces ECM deposition in NPDFs, as indicated by an increase in the expression of collagen and fibronectin. Therefore, we investigated whether 1,25(OH)_2_D_3_ inhibits ECM production in NPDFs. Treatment with 1,25(OH)_2_D_3_ decreased TGF-β1-induced fibronectin mRNA and protein levels (Fig. 2A,B). As shown with immunofluorescence observation (Fig. 2C), 1,25(OH)_2_D_3_ suppressed fibronectin expression in TGF-β1-stimulated NPDFs. In addition, we used the Sircol collagen assay to determine Total soluble collagen levels in the supernatant of cultured NPDFs. Induction of secreted collagen by TGF-β1 was completely abolished by treatment with 1,25(OH)_2_D_3_ (Fig. 2D). These results suggest that 1,25(OH)_2_D_3_ inhibits ECM production triggered by TGF-β1, directly reducing expression of fibronectin and synthesis of collagen in NPDFs.

### 1,25(OH)_2_D_3_ abrogates myofibroblast differentiation and collagen production via reducing phosphorylation of smad2/3 mediated by binding to vitamin D receptors in nasal polyp-derived fibroblasts

**Figure 3:**
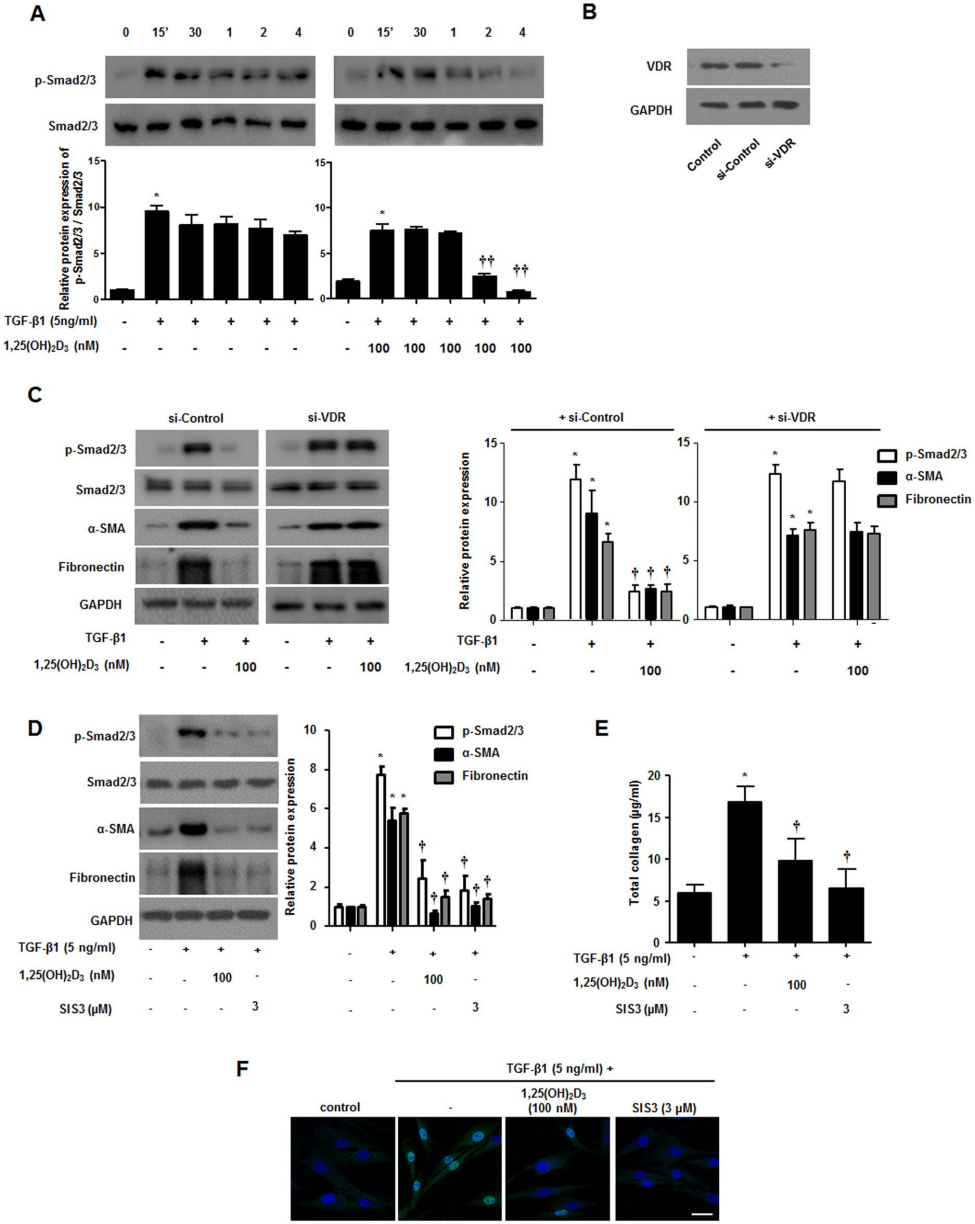
1,25(OH)_2_D_3_ inhibits phosphorylation of smad2/3 in nasal polyp-derived fibroblasts. Nasal polyp-derived fibroblasts were treated with TGF-β1 and/or 1,25(OH)_2_D_3_ up to 4 hours. (**A**) Expression levels of phosphorylated Smad2/3 were determined by Western blotting. (**B**) Specific *VDR* siRNA (10 nM) was transfected in NPDFs and the inhibitory effect of *VDR* siRNA was confirmed by Western blotting. (**C**) Transfected NPDFs with *VDR* siRNA were treated with or without 1,25(OH)_2_D_3_ (100 nM) for 1 hour and cells were stimulated with TGF-β1 (5 ng/ml) for 4 hours and 72 hours. Phosphorylated Smad2/3 and α-SMA, fibronectin was determined by Western blotting. (**D**) NPDFs were treated with TGF-β1 (5 ng/ml) and/or 1,25(OH)_2_D_3_ (100 nM) or SIS3 (a Smad3-specific inhibitor, 3 μM). Expression levels of phosphorylation of Smad2/3 (4 hours) and α-SMA and fibronectin (72 hours) were determined by Western blotting. Expression levels of the housekeeping GAPDH protein were utilized as internal controls. A representative experiment and quantitative determination of protein levels are shown. (**E**) The amount of total soluble collagen in culture media was quantified by the Sircol collagen assay. (**F**) Representative fluorescein immunocytochemistry for fibronectin (green) with nuclear DAPI (blue). Scale bar = 50 μm. All data are presented as mean ± SEM. Four primary cell lines from different donors were used. All experiments were performed in at least triplicate and repeated at least three times. *p < 0.05 vs. control, ^†^p < 0.05, ^††^p < 0.01 vs. TGF-β1.

Smad2/3 signaling is a critical pathway induced by TGF-β1. Smad2/3 phosphorylation and translocation into the nucleus regulate α-SMA and fibronectin through binding to pro-fibrotic genes^20^. We investigated whether 1,25(OH)_2_D_3_ could block the Smad2/3 signaling pathway in NPDFs. TGF-β1 treatment induced phosphorylation of smad2/3 from 15 minutes to 4 hours and 1,25(OH)_2_D_3_ inhibited TGF-β1-induced phosphorylation after 2 hours (Fig. 3A). To determine whether this inhibition mechanism is mediated by the vitamin D receptor (VDR), we confirmed knockdown of *VDR* using siRNA (Fig. 3B) and the effects of 1,25(OH)_2_D_3_ via Western blotting. The effects of 1,25(OH)_2_D_3_ significantly inhibited the phosphorylation of Smad2/3 and upregulated α-SMA and fibronectin through the formation of complex with VDR in NPDFs (Fig. 3C). We also found that enhancement of α-SMA and fibronectin expression and overproduction of total soluble collagen by TGF-β1 was suppressed in NPDFs after direct treatment with SIS3, a Smad2/3-specific inhibitor (Fig. 3D and E), similar to that of treatment with 1,25(OH)_2_D_3_. Confocal microscopy to identify translocation of p-Smad2/3 (Fig. 3F) displayed that 1,25(OH)_2_D_3_ markedly blocked translocation of p-Smad2/3 from the cytoplasm to the nucleus. Taken together, these data suggest that 1,25(OH)_2_D_3_ has anti-fibrotic activity via Smad2/3, which is a downstream molecule in the TGF-β1 signaling pathway in NPDFs.

### 1,25(OH)_2_D_3_ antagonizes TGF-β1-induced acetylation of histone H3 in nasal polyp-derived fibroblasts

**Figure 4:**
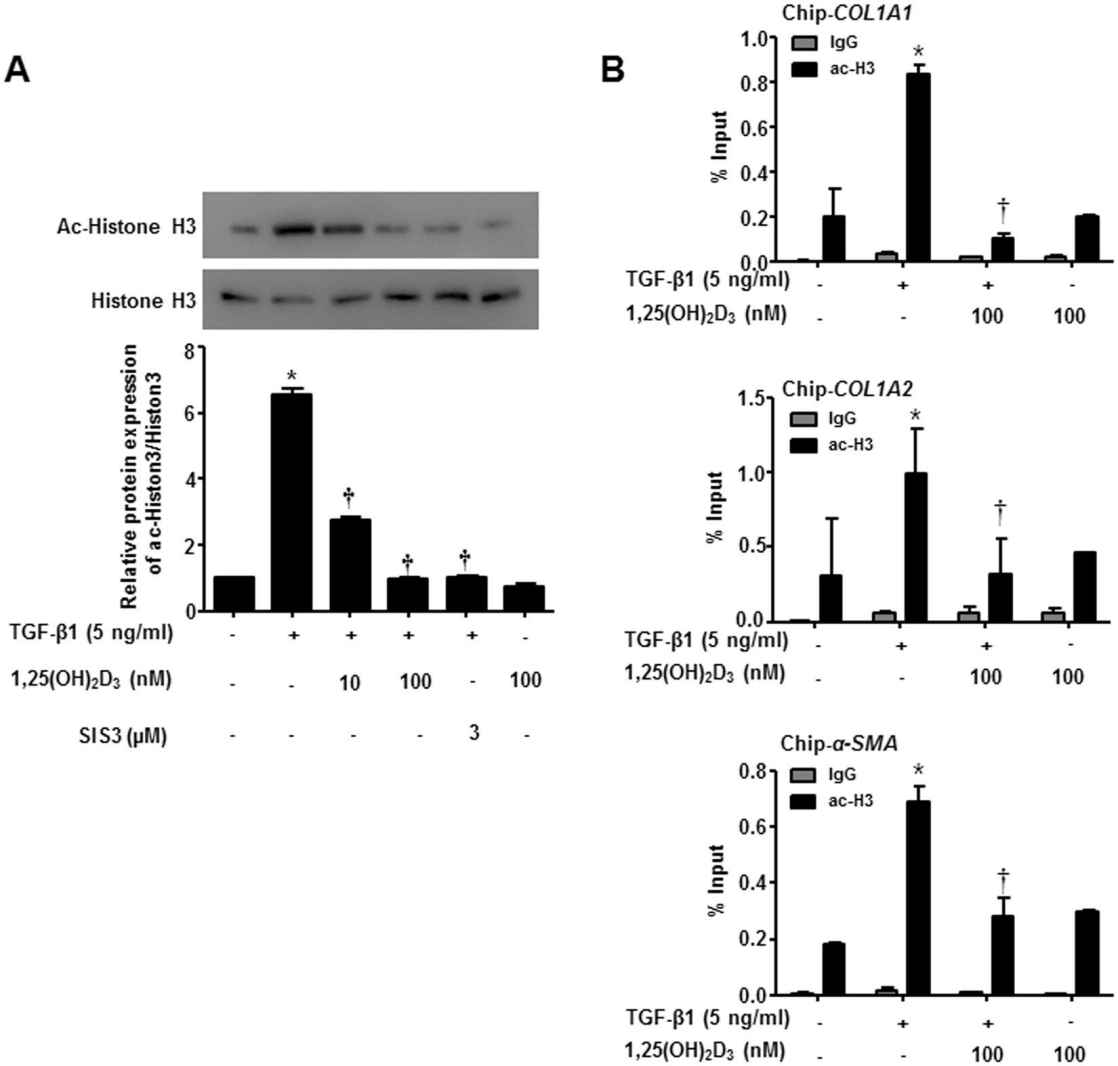
1,25(OH)_2_D_3_ decreases TGF-β1/Smad signaling pathway via reducing Histone 3 acetylation in nasal polyp-derived fibroblasts. Nasal polyp-derived fibroblasts were treated with TGF-β1 and/or 1,25(OH)_2_D_3_ or 1,25(OH)_2_D_3_ alone or 4 hours. (**A**) Expression levels of acetylated-histone 3 were determined by Western blotting. Expression of histone 3 was utilized as the internal control. A representative experiment and quantitative determination of protein levels are shown. (**B**) The mRNA of *COL1A1*, *COL1A2* and *α-SMA* promoter and its adjacent regions precipitated by acetyl-H3 were quantified by ChIP assay. All data are presented as mean ± SEM. Four primary cell lines from different donors were used. All experiments were performed in at least triplicate and repeated at least three times. *p < 0.05 vs. control, ^†^p < 0.01 vs. TGF-β1.

1,25(OH)_2_D_3_ reportedly hinders ECM synthesis by TGF-β1/Smad and chromatin rearrangement in TGF-β1-treated hepatic stellate cells^21^, which we determined whether 1,25(OH)_2_D_3_ could block transcription of α-SMA and collagen COL1A1 and COL1A2 via interference with acetylated histone H3. In agreement with the previous document, hyperacetylation of histone H3 was stimulated by TGF-β1 treatment, but cells treated with 1,25(OH)_2_D_3_ significantly abrogated histone H3 hyperacetylation, blocking activation of TGF-β1/Smad signaling at the indicated dose (p < 0.05, Fig. 4A). Interestingly, a Smad3-specific inhibitor, SIS3, also reduced enhanced histone H3 acetylation by TGF- β1. NPDFs treated with vitamin D only suppressed acetylation of histone H3 slightly. Furthermore, via ChIP-qPCR assay, we found that TGF-β1 induced TGF-β1/SMAD binding at the regulatory region of *α-SMA, COL1A1* and *COL1A2*, triggering H3 hyperacetylation, and thus enhancing transcription of *α-SMA*, *COL1A1* and *COL1A2*. On the other hand, 1,25(OH)_2_D_3_ blocked upregulated transcription of tissue remodeling genes by TGF-β1, suggesting that 1,25(OH)_2_D_3_ modulates histone H3 hyperacetylation, leading to inhibition of functional activity including myofibroblast differentiation and overproduction of ECM in NPDFs (Fig. 4B).

### 1,25(OH)_2_D_3_ inhibits TGF-β1-induced contractile activity and cell migration in nasal polyp-derived fibroblasts

**Figure 5:**
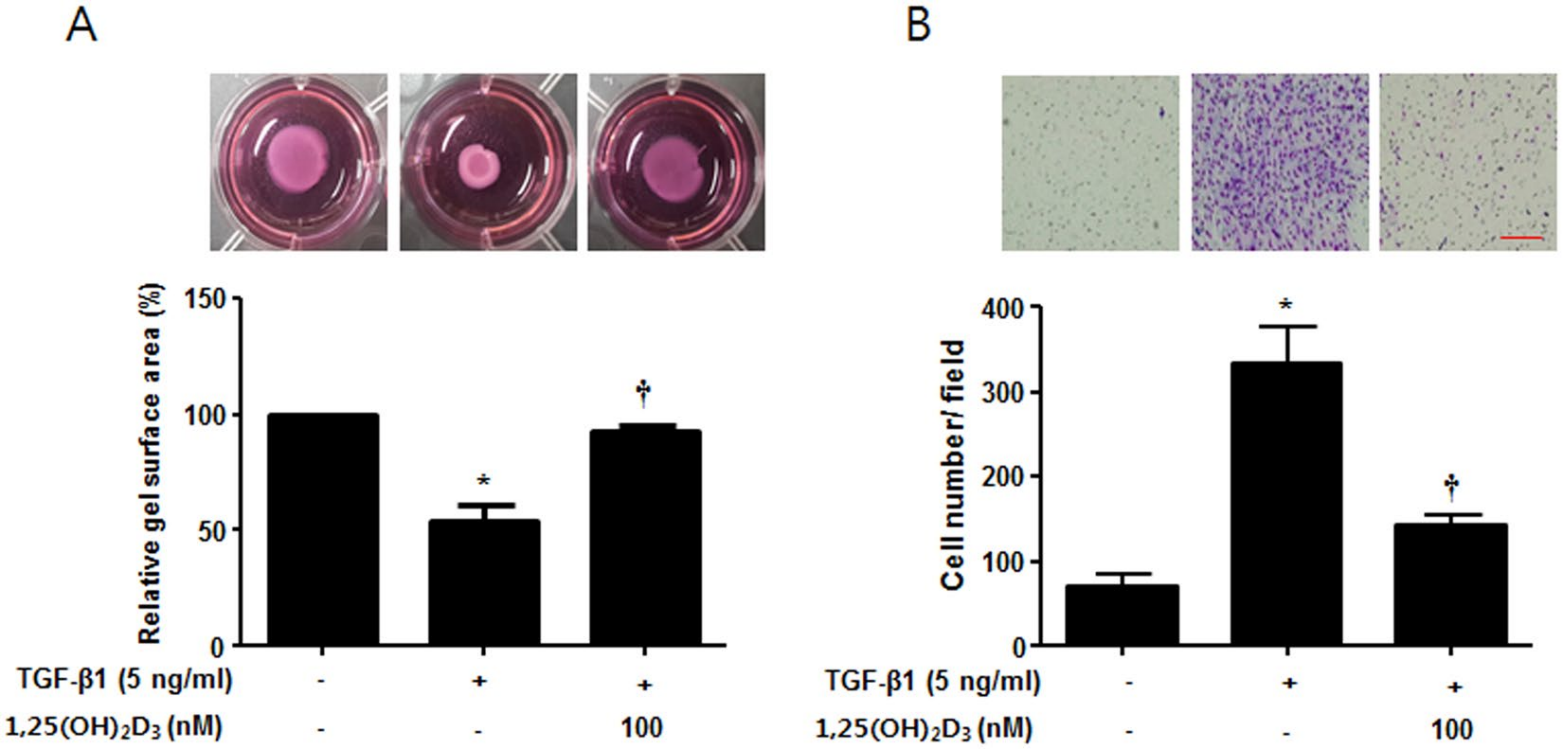
1,25(OH)_2_D_3_ decreases TGF-β1-induced collagen gel contraction and cell migration in nasal polyp-derived fibroblasts. Nasal polyp-derived fibroblasts were treated with TGF-β1 and/or 1,25(OH)_2_D_3_ for 72 hours. (**A**) Contractile activity was assessed by collagen gel contraction assay. These pictures show the results of one experiment; the contraction area was measured using an Image J analyzer. (**B**) Cell migration was assessed by Transwell migration assay. These pictures show one experimental result; cell number was measured using an Image J analyzer. All data are presented as mean ± SEM. Four primary cell lines from different donors were used. All experiments were performed in at least triplicate and repeated at least three times. *p < 0.05 vs. control, ^†^p < 0.01 vs. TGF-β1.

Myofibroblasts play a central role in repair of wound tissues through their capacity to produce strong contractile forces and recruit cell migration^22^. Thus, we examined whether 1,25(OH)_2_D_3_ regulates contractile activity and migration of myofibroblast using collagen gel contraction and Transwell migration assays. TGF-β1 stimulated contraction of the collagen gel to 54.0 ± 12.1% of the initial area 24 hours after TGF-β1 stimulation, as previously described^23, 24^, and 1,25(OH)_2_D_3_ significantly inhibited TGF-β1-induced contraction of collagen gel by 92.3 ± 4.5% of the initial area (p < 0.05). In addition, migration activity of NPDFs via TGF-β1 stimulus was significantly reduced by 1,25(OH)_2_D_3_ from 333.3 ± 76.4 to 143.3 ± 20.8 (p < 0.05) (Fig. 5A,B). These results suggest that 1,25(OH)_2_D_3_ inhibits the functional activity of myofibroblasts by regulating decreased contractile and cell migration activities.

### 1,25(OH)_2_D_3_ inhibits expression levels of α-SMA and fibronectin and total collagen production in *ex vivo* organ culture of nasal polyps

**Figure 6:**
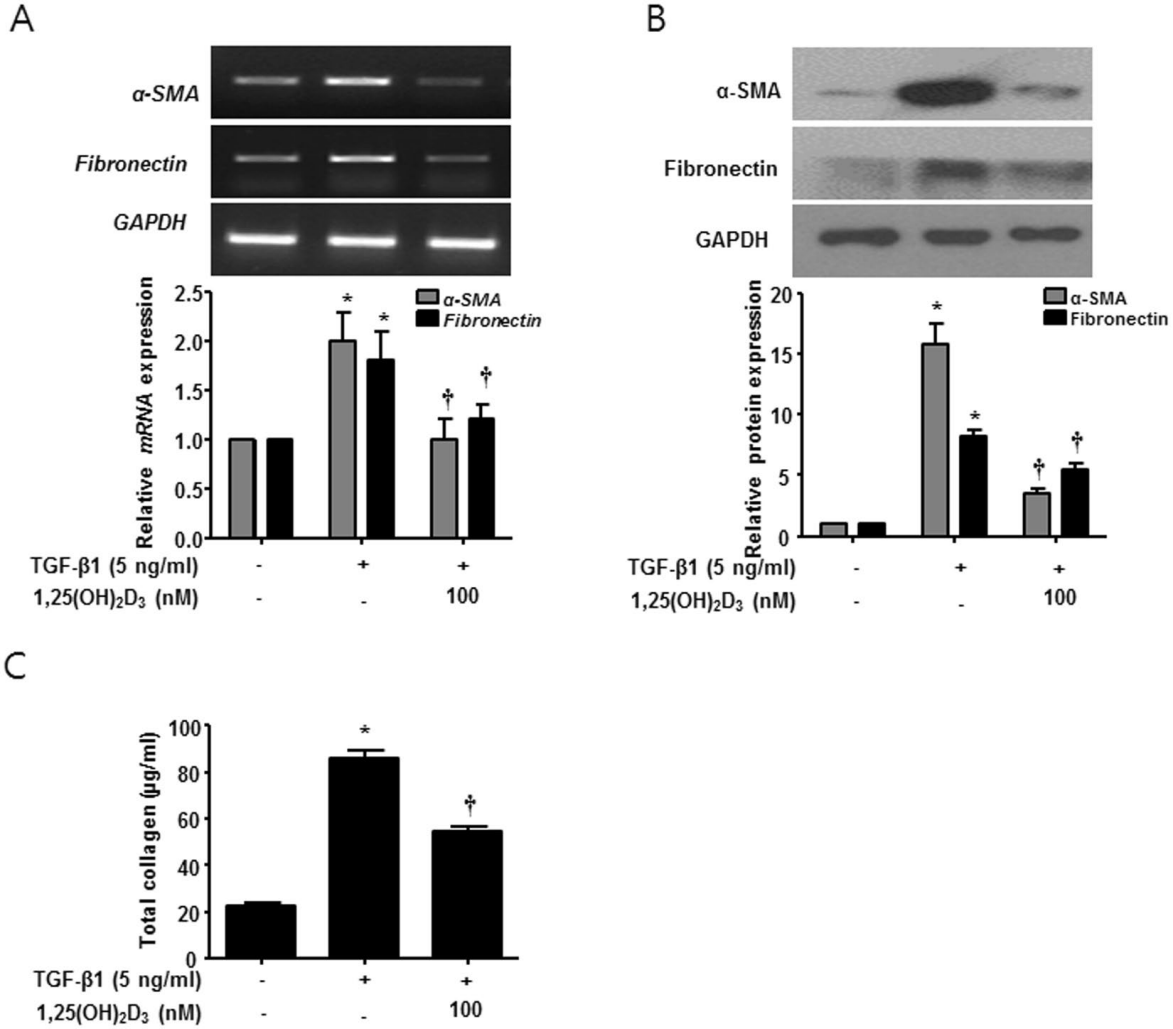
1,25(OH)_2_D_3_ decreases TGF-β1-induced collagen production in *ex vivo* organ culture of nasal polyps. Nasal polyps were stimulated with TGF-β1 and/or 1,25(OH)_2_D_3_ for 72 hours. (**A**,**B**) Expression levels of α-SMA and fibronectin mRNA and protein were determined by semi-quantitative RT-PCR analysis and Western blot analysis. Expression of housekeeping GAPDH was utilized as an internal control. A representative experiment and quantitative determination of α-SMA mRNA and protein levels are shown. (**C**) The amount of total soluble collagen in culture media was quantified using the Sircol assay. All data are presented as mean ± SEM. Four nasal polyp specimens from different donors were used. All experiments were performed in at least triplicate and repeated at least three times. *p < 0.05 vs. control, ^†^p < 0.01 vs. TGF-β1.

To confirm the inhibitory effects of 1,25(OH)_2_D_3_ on protein expression of α-SMA and fibronectin and collagen production in human tissues, we performed *ex vivo* organ culture of nasal polyps. 1,25(OH)_2_D_3_ significantly inhibited expression of α*-*SMA and fibronectin and total collagen production in *ex vivo* organ culture of nasal polyps treated with TGF-β1 (Fig. 6). These results strongly suggest that 1,25(OH)_2_D_3_ can be used as a potent therapeutic agent for myofibroblast differentiation and ECM production in NPDFs under the tissue remodeling conditions induced by TGF-β1.

## Discussion

**Figure 7:**
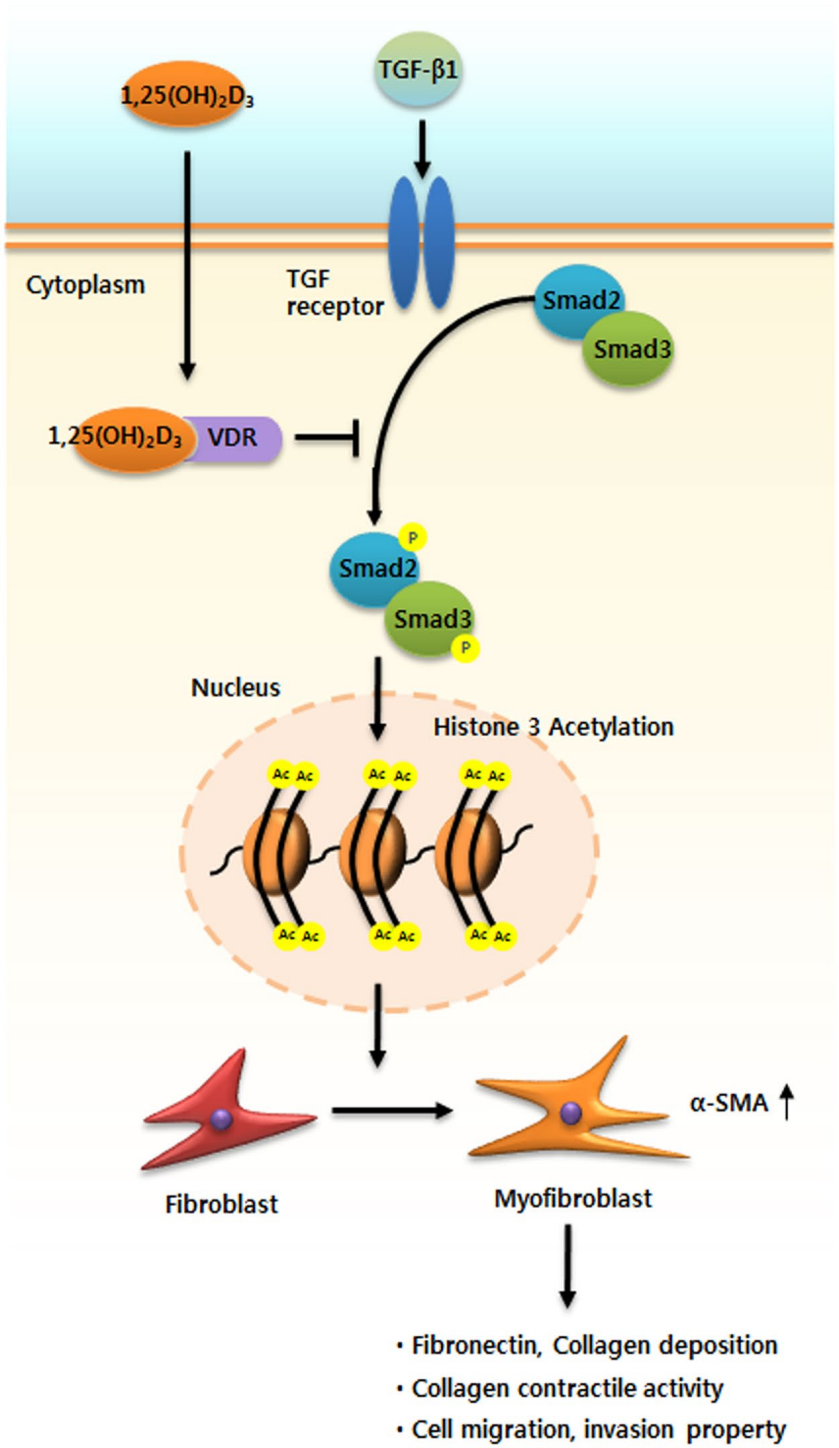
Role of 1,25(OH)_2_D_3_ in myofibroblast differentiation and extracellular matrix deposition in TGF-β1-induced nasal polyp-derived fibroblasts and nasal *ex vivo* organ culture. 1,25(OH)2D3 suppressed myofibroblast differentiation and extracellular matrix production by reducing acetylation of histone 3 through inactivation of the Smad2/3 signaling pathway associated with vitamin D receptors, resulting in prevention of collagen contractile activity and cell migration in the upper airway under TGF-β1 stimulus.

In this study, we evaluated the anti-tissue remodeling role and underlying mechanisms of vitamin D action in the formation of nasal polyps (Fig. 7). Our results demonstrated that treatment with vitamin D reduced expression of α-SMA and fibronectin and total collagen production and functionally suppressed collagen contraction and cell migration in TGF-β1-induced NPDFs and *ex vivo* organ culture of nasal polyps. Taken together, these results suggest that vitamin D inhibited significant fibrotic alterations associated with myofibroblast differentiation and excessive ECM production in NPDFs stimulated by TGF-β1 through Smad2/3 signaling pathways.

Nasal polyp formation is a difficult and recalcitrant condition, with an unclear etiology and frequent recurrence in clinical rhinology^25^. Many studies have demonstrated that TGF-β1 is the prime stimulator of fibroblast activation and that it can induce activation and differentiation of fibroblasts into myofibroblasts expressing α-SMA. TGF-β1 promotes high levels of ECM deposition, which can lead to airway tissue remodeling^26–28^. We previously demonstrated that both mRNA and protein expression levels of α-SMA and TGF-β1 were markedly higher in nasal polyp tissues than in normal inferior turbinate tissues, suggesting that tissue remodeling is involved in nasal polyp formation^23^.

Vitamin D, a secosteroid hormone, has recently attracted considerable attention due to its wide range of biological activities in several organs^6^. Wang *et al*. demonstrated significantly low serum levels of vitamin D in CRSwNP patients^29^. Furthermore, vitamin D provides significant protection against human nasal polyp formation by reducing the size of nasal polyps and relieving the symptoms and signs of nasal polyps^30^. Anti-fibrotic and tissue remodeling activities of active vitamin D counteract pro-fibrotic TGF-β1, inhibiting myofibroblast activation and suppressing α*-*SMA expression in renal interstitial fibroblasts and lung fibroblasts^7, 18^. However, the role of vitamin D in the pathogenesis of nasal polyp formation remains largely unexplored. Specifically, it is unclear whether vitamin D affects the essential functions of myofibroblast differentiation and ECM production in NPDFs. We hypothesized that vitamin D influences pro-fibrotic processes in NPDFs and *ex vivo* organ culture of nasal polyps. In agreement with previous reports, we showed that TGF-β1 greatly stimulated α*-*SMA expression, a specific marker of myofibroblast differentiation, whereas vitamin D counteracted this effect at both the transcript and protein levels. Similarly, vitamin D-treated NPDFs displayed diminished TGF-β1-related expression of fibronectin and production of total collagen. Taken together, these results indicate that vitamin D suppresses myofibroblast differentiation and ECM production in NPDFs stimulated by TGF-β1.

TGF-β1 induces myofibroblast differentiation in part via the Smad2/3 signaling pathway^28, 31^. The activated complex is phosphorylated and forms a heteromeric receptor complex with TGF-βRI after active TGF-β1 binds to TGF-βRII; it subsequently phosphorylates Smad2 and Smad3, which binds to Smad4, followed by translocation into the nucleus where the complex increases *α-SMA* gene transcription. A previous study showed that vitamin D supplementation suppresses renal fibrosis through stimulation of vitamin D receptor-mediated transcription, which inhibits TGF-β1-Smad signal transduction^32^. According to a recent report, pro-fibrotic gene expression is mediated by Smad translocation to the nucleus and chromatin remodeling under response of TGF-β1, 1,25(OH)_2_D_3_ subsequently blocks acetylation of histone H3 in TGF-β1-induced hepatic stellate cells^21^. Moreover, we previously proposed that the TGF-β1/Smad2/3 signaling pathways are involved in myofibroblast differentiation and ECM production in NPDFs^23^. Thus, we also used Western blot analysis, ChIP-qPCR, confocal microscopy, and measurement of total collagen to explain that vitamin D is associated with the TGF-β/Smad signaling pathway in TGF-β1-induced NPDFs. Interestingly, we found that the phosphorylation and translocation of Smad2/3 were significantly decreased by treatment with vitamin D in TGF-β1-induced NPDFs. In addition, knockdown of VDR-NPDFs showed no effect on p-Smad2/3 and tissue remodeling-mediated protein expression. Furthermore, SIS3 (a Smad3-specific inhibitor) treatment in TGF-β1-induced NPDFs caused downregulation of α*-*SMA and fibronectin protein expression and collagen production, similar to the effect of vitamin D. We also determined that vitamin D reduced histone H3 hyperacetylation, further compromising transcription of *COL1A1*, *COL1A2* and *α-SMA* genes. Taken together, these data suggest that vitamin D ameliorates fibroblast differentiation into myofibroblasts and ECM production via Smad2/3-mediated processes in NPDFs.

Activated fibroblasts play a critical role in the wound repair and scarring processes that trigger wound contraction at the site of damage, to which fibroblasts begin to migrate^22^. Kumar *et al*. described that the increased contraction and migration induced by TGF-β1 were significantly reduced by mitomycin-C in human nasal mucosal fibroblasts^33^. In the present study, we showed that treatment with vitamin D suppressed enhancement of cellular functions such as gel contraction and migration of NPDFs after treatment with TGF-β1, suggesting that vitamin D has therapeutic potential for nasal polyps.

Since an *in vivo* model of nasal polyps has not been established, previous reports used nasal *ex vivo* organ culture to study the physiology and pathology of nasal polyps, providing an accessible means to mimic *in vivo* conditions, including cell-to-cell contact, cell-to-matrix integrity, and maintenance of three-dimensional structures^34, 35^. In our study, we confirmed the inhibitory effects of vitamin D in *ex vivo* organ culture of nasal polyp tissue, positively reducing the TGF-β1-mediated effects on expression of α*-*SMA and fibronectin and production of collagen.

Based on the current evidence, we propose a model that supports the anti-tissue remodeling activity of vitamin D via mediating suppression of TGF- β1/Smad2/3 signaling pathways and downregulation of histone H3 (Fig. 7). Herein we described a mechanism by which vitamin D preferentially acts as an anti-tissue remodeling agent under TGF-β1-triggered conditions; for example, under constitutively enhanced myofibroblast differentiation and production of ECM in NPDFs. In this model, vitamin D functions as a promising therapeutic agent by inhibiting the expression of α*-*SMA, eventually leading to suppression of ECM production. Vitamin D appears to have anti-tissue remodeling activities related to its modulation of myofibroblast differentiation and ECM production in NPDFs under TGF-β1 stimulation via blockade of TGF-β1-Smad2/3 signaling pathways and hyperacetylation of histone 3, which could contribute to the treatment and prevention of nasal polyps.

## Materials and Methods

### Nasal polyp-derived fibroblast culture and treatment

Eight patients with nasal polyps were recruited from the Department of Otorhinolaryngology, Korea University Medical Center, and nasal polyp tissues were obtained during surgical procedures. All patients were nonsmokers and had not been treated with oral or topical corticosteroids or antibiotics for at least 4 weeks before surgery. There were no known allergies, asthma, or aspirin sensitivities among the patients. This study was approved by the Korea University Medical Center Institutional Review Board. Written informed consent was obtained from all subjects, and this study was conducted according to the principles of the Declaration of Helsinki.

Isolation and confirmation of NPDFs were conducted as previously described^36^. Cells were cultured in Dulbecco’s modified Eagle’s medium (DMEM; Invitrogen, Grand Island, NY) containing 10% heat-inactivated fetal bovine serum (FBS; Invitrogen), 10,000 units/mL penicillin, and 10,000 μg/mL streptomycin (Invitrogen) at 37 °C in a 5% CO_2_ incubator. NFDFs at the third to seven passages were used in the following experiments.

NPDFs were treated with human recombinant TGF-β1 (R&D Systems, Minneapolis, MN) and/or 1,25(OH)_2_D_3_ (Sigma-Aldrich Co., St. Louis, MO) to evaluate their inhibitory effects on TGF-β1-induced myofibroblast differentiation and collagen production.

### Semi-quantitative reverse transcription-polymerase chain reaction (RT-PCR)

Total RNA was extracted using the TRIzol RNA isolation protocol, and the first-strand cDNA was synthesized using 2 μg RNA in 20 μL of reaction buffer with MMVL reverse transcriptase (Promega, Madison, WI) according to the manufacturer’s instructions. PCR was performed using the following primers: *α-SMA* (sense sequence 5′-GGTGCTGTCTCTCTATGCCTCTGG A-3′ and antisense sequence 5′-CCCAT CAGGCAACTCGATACTCTTC-3′; 321 bp), *fibronectin* (sense sequence 5′-GGATGCTCC TGCTGTCAC-3′ and anti sense sequence 5′-CTGTTTGATCTGGACCTGCAG-3′), *glyceraldehyde-3-phosphate dehydrogenase* (*GAPDH*; sense sequence 5′-GTGGATATTGTTGCCATCAATGACC-3′ and anti sense sequence 5′-GCCCCAGCCTTCTTCATG GTGGT-3′, 271 bp). The PCR samples were electrophoresed on 1% agarose gels in TBE buffer (89 mM Tris-base pH 7.6, 89 mM boric acid, 2 mM EDTA). The gels were stained with ethidium bromide (10 μg/ml) and photographed using a 280-nm UV light box. The gel images were digitally captured with a CCD camera. Densitometry values were measured at each cycle sampling using the Image J. Expression levels of the target mRNAs were normalized to *GAPDH*.

### Western blotting analysis

NPDFs were lysed using PRO-PREP^TM^ protein extraction solution (iNtRON Biotechnology, Seongnam, Korea). Cell lysates were subjected to SDS-PAGE and transferred to polyvinylidene difluoride membranes (Millipore Inc., Billerica, MA). Membranes were probed with primary antibodies overnight at 4 °C. The primary antibodies used included the followings: anti-α-SMA (Chemicon, Millipore Inc., Billerica, MA), anti-acetyl H3 (Cell Signaling), anti-histone H3, anti-fibronectin, anti-phospho-smad2/3, anti-total smad2/3, and glyceraldehyde-3-phosphate dehydrogenase (Santa Cruz Biotechnology, Inc., Santa Cruz, CA). Membranes were washed for 5 minutes three times, incubated with peroxidase-conjugated anti-mouse or anti-rabbit antibodies (Vector Laboratories, Burlingame, CA) for 1 hour, and washed for 5 minutes three times. Protein expression was detected using Amersham ECL Western Blotting Substrate (GE Healthcare, Little Chalfont, Buckinghamshire, UK).

### Transient knockdown of *VDR* with siRNA

Transient knockdown was carried out with *VDR* siRNA (Bioneer, Daejeon, Korea) at a concentration of 10 nM. Transfection was performed in NPDFs using Lipofectamine 2000 (Invitrogen) for 18 hours. The knockdown effect of VDR protein was confirmed via Western blot.

### Measurement of total collagen amount

The Sircol soluble collagen assay (Biocolor Ltd., Newtownabbey, UK) was used to quantify total soluble collagen. Briefly, collected supernatant was mixed with Sirius Red dye for 30 minutes, and then the pellets were dissolved in 0.5 M sodium hydroxide and vortexed. Absorbance was measured at 550 nm using a fluorescence microplate reader (SpectraMax Plus 384, Molecular Devices, San Francisco, CA).

### Immunofluorescence staining

NPDFs were fixed with 4% paraformaldehyde and permeabilized with 0.2% TritonX-100 in 1% bovine serum albumin for 10 minutes. After blocking in 3% bovine serum albumin for 1 hour, cells were incubated with primary antibody (anti-α*-*SMA (1:100), anti-fibronectin (1:100), or anti-p-Smad2/3 (1:100)) in a moist, 4 °C chamber overnight, washed, and then incubated for 1 hour with goat anti-mouse Alexa 488 (Invitrogen) or goat anti-rabbit Alexa Fluor 555 (Invitrogen) secondary antibodies at room temperature. Each stained NPDF was captured and visualized using confocal laser scanning microscopy (LSM700, Zeiss, Oberkochen, Germany). Cell nuclei were double-stained with DAPI (Vectashield mounting medium, Vector Laboratories).

### Chromatin immunoprecipitation assay

NPDFs were treated with TGF-β1 and/or 1,25(OH)_2_D_3_ for 4 hours. Cells were harvested for the Chip assay from Upstate (EZ ChIP kit, Millipore Inc. Billerica, MA) according to the manufacturer’s instructions. Briefly, after fixation, nuclei from NPDFs were isolated, lysed, sheared with sonication. Afterward, sheared DNA was incubated with a normal mouse immunoglobulin G (Upstate), mouse anti-Ace-H3 antibody (Cell Signaling). After immunoprecipitation, the cross-linked DNA was released, reversed, and then purified with the provided spin column. *Col1A1*, *Col1A2* and *α-SMA* expression were examined by quantitative PCR, performed on Quantstudio3 (Applied Biosystems, Foster City, CA) using Power SYBR Green PCR Master Mix (Applied Biosystems, Foster City, CA) followed by initial denaturing step at 95 °C for 15 seconds, 50 cycles of denaturing at 95 °C for 5 seconds. and annealing at 60 °C for 30 seconds. The sequence of *Col1A1, Col1A2* and *α-SMA* primers was provided in previous documents^20, 22^. Enrichment of ChIP-DNA was defined as the ratio of the PCR product of ChIP DNA to the input DNA.

### Collagen gel contraction assay

NPDFs (3 × 10^5^) were mixed with reconstituted collagen solution consisting of eight volumes of rat-tail tendon collagen type I (BD Bioscience, Bedford, MA) to one volume of reconstituted buffer (260 mM NaHCO_3_, 200 mM HEPES, 50 mM NaOH) on ice. Then, 500 μl of the reconstituted collagen mixture was placed in each well of a 24-well tissue culture plate and allowed to polymerize at 37 °C for 1 hour. After polymerization, the gels were gently transferred to six-well culture plates containing 1.5 mL serum-free-DMEM with TGF-β1 (5 ng/mL) and/or 1,25(OH)_2_D_3_ (100 nM). The gels were then incubated at 37 °C in a 5% CO_2_ atmosphere for 3 days. The area of each gel was measured using an Image J analyzer (NIH, Bethesda, MA). Data are expressed as the percentage of area compared with the initial gel area.

### Cell migration assay

For transwell migration assays, NPDFs (1.5 × 10^4^) were seeded onto Transwell chambers (Corning Life Sciences, MA) and cultured for 48 hours in DMEM containing 10% FBS, TGF-β1 (5 ng/mL), and/or 1,25(OH)_2_D_3_ (100 nM). Cells on the upper surface of the membrane were removed using cotton swabs, and then the cells on the lower surface of the membrane were stained using Diff-Quik staining (Sysmex, Kobe, Japan). Images of stained cells from five selected views were captured under microscopy at 200x magnification (Olympus BX51; Olympus, Tokyo, Japan).

### *Ex vivo* organ culture of nasal polyps

*Ex vivo* organ culture of nasal polyps was performed as described previously by Cho *et al*.^33^. Briefly, 2 to 3 mm^3^ of nasal polyp tissues were washed 3 times with phosphate- buffered saline (PBS) and rinsed with culture medium composed of DMEM, 2% FBS, 100 U/mL penicillin (Invitrogen), 100 mg/mL streptomycin (Invitrogen), and 0.25 mg/mL fungizone. The rinsed tissue fragments were placed on a pre-hydrated 10 × 10 × 61-mm gelatin sponge (Spongostan, Johnson & Johnson, San Angelo, TX) with the mucosa side facing up and the submucosa side facing down. Tissue fragments were placed onto 6-well plates filled with 1.5 mL of culture medium per well such that the mucosa was above the liquid phase. Nasal polyps were stimulated with TGF-β1 (5 ng/mL) and/or 1,25(OH)_2_D_3_ (100 nM) for 72 hours.

### Statistical analysis

All data are presented as mean ± SEM. The statistical significance of differences between groups was assessed by one-way analysis of variance for factorial comparisons and by Tukey’s multiple comparison tests for multiple comparisons. All experiments were performed in at least triplicate and repeated at least three times.

## Electronic supplementary material

Fig. S1.
