# Supplementary material for: Vitamin D attenuates myofibroblast differentiation and extracellular matrix accumulation in nasal polyp-derived fibroblasts through smad2/3 signaling pathway
Source: Sci Rep. 2017 Aug 4;7:7299. doi: 10.1038/s41598-017-07561-6 (PMC5544725; doi:10.1038/s41598-017-07561-6)
Supplement: Supplementary file 1 — Fig. S1. [file 41598_2017_7561_MOESM1_ESM.doc]

**Vitamin D attenuates myofibroblast differentiation and extracellular matrix accumulation in nasal polyp**-**derived fibroblasts through the smad2/3 signaling pathway**

Seoung Ae Lee^1,4^, Hyun-Woo Yang^2^, Ji-Young Um^2^, Jae-Min Shin^1,3^, Il-Ho Park^1,2,3^, Heung‑Man Lee^1,2,3,4^

^1^Institute for Medical Devices Clinical Trial Center, Korea University Guro Hospital, Korea University, College of Medicine, Seoul, Korea, ^2^Biomedical Science, Korea University, College of Medicine, ^3^Department of Otorhinolaryngology-Head and Neck Surgery, Korea University, College of Medicine, Seoul, Korea, ^4^Research-Driven Hospital, Korea University Guro Hospital, Korea University, College of Medicine, Seoul, South Korea

Corresponding author:

Heung-Man Lee, MD, PhD

Department of Otorhinolaryngology - Head and Neck Surgery

Guro Hospital, Korea University College of Medicine

80 Guro-dong, Guro-gu, Seoul 152-703, South Korea

Telephone: 82-2-2626-3185 Fax: 82-2-868-0475

E-mail: [lhman@korea.ac.kr](mailto:lhman@korea.ac.kr)


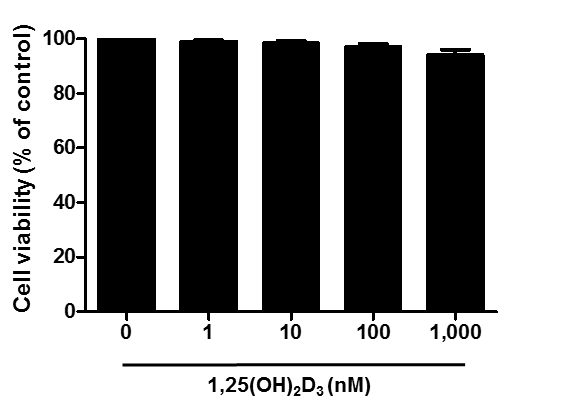


**Fig. S1. Effect of 1,25(OH)_2_D_3_ at different concentrations (0-1000nM) on the viability in NPDFs.** For the cell viability assay, NPDFs were seeded on 96 well plates (1x10^4^ cells/100 μl), followed by TGF-β1(5 ng/ml) and/or 1,25(OH)_2_D_3_ (0 - 10,000 nM) or 1,25(OH)_2_D_3_ alone treatment. After 72 hours, cell culture medium was replaced by MTT working solution (Sigma- **Aldrich Co**), followed by a 4 hours incubation at 37 ˚C in 5% CO2 incubator. After MTT working solution was removed and resolved with dimethyl sulfoxide, the absorbances at 590 were detected using by a fluorescence microplate reader (SpectraMax Plus 384, Molecular Devices, San Francisco, CA). All data are presented as mean **±**SEM. All experiments were performed in at least triplicate and were repeated at least three times using independent cell cultures.
